# Genotype-Independent Transmission of Transgenic Fluorophore Protein by Boar Spermatozoa

**DOI:** 10.1371/journal.pone.0027563

**Published:** 2011-11-16

**Authors:** Wiebke Garrels, Stephanie Holler, Ulrike Taylor, Doris Herrmann, Christina Struckmann, Sabine Klein, Brigitte Barg-Kues, Monika Nowak-Imialek, Christine Ehling, Detlef Rath, Zoltán Ivics, Heiner Niemann, Wilfried A. Kues

**Affiliations:** 1 Friedrich-Loeffler-Institut, Mariensee, Germany; 2 Max Delbrück Center for Molecular Medicine, Berlin, Germany; University of Birmingham, United Kingdom

## Abstract

Recently, we generated transposon-transgenic boars (*Sus scrofa*), which carry three monomeric copies of a fluorophore marker gene. Amazingly, a ubiquitous fluorophore expression in somatic, as well as in germ cells was found. Here, we characterized the prominent fluorophore load in mature spermatozoa of these animals. Sperm samples were analyzed for general fertility parameters, sorted according to X and Y chromosome-bearing sperm fractions, assessed for potential detrimental effects of the reporter, and used for inseminations into estrous sows. Independent of their genotype, all spermatozoa were uniformly fluorescent with a subcellular compartmentalization of the fluorophore protein in postacrosomal sheath, mid piece and tail. Transmission of the fluorophore protein to fertilized oocytes was shown by confocal microscopic analysis of zygotes. The monomeric copies of the transgene segregated during meiosis, rendering a certain fraction of the spermatozoa non-transgenic (about 10% based on analysis of 74 F1 offspring). The genotype-independent transmission of the fluorophore protein by spermatozoa to oocytes represents a non-genetic contribution to the mammalian embryo.

## Introduction

DNA Class II transposons have been successfully used for transgenesis and insertional mutagenesis in invertebrates [1], [2] and transposase-catalyzed transgenesis in vertebrates has been initiated by reactivation of the *Sleeping Beauty* (SB) transposon system [3]. Several other transposases, such as *Tol1, Tol2* and *piggyBac* have been shown to be functional for transgenesis in fish, frogs, birds and rodents [4]–[16]. The SB system has gained special interest for successful gene transfer in the pig [17]-[20], which is an important large animal model for biomedicine [21], [22]. Drawbacks of classical methods for transgenesis [23]–[26] can be overcome by utilizing transposase-catalyzed gene delivery, as it increases the efficiency of chromosomal integration, facilitates single-copy (monomeric) insertion events and provides predictable transgene expression patterns.

Recently, we had shown that cytoplasmic plasmid injection (CPI) of zygotes [27], [28] with plasmids encoding components of the SB system is a highly efficient method for porcine transgenesis [17], [18]. Improvements of current technologies to modify the genome of pigs will be instrumental for the further development of this important biomedical model [21], [22], [24], [26], [29]. Own data revealed that SB-transposon transgenic founder boars (F0) showed expression of the fluorescent Venus reporter in nearly all cell types [17], [18], including a prominent Venus load in mature spermatozoa. To the best of our knowledge, in none of the other transposon transgenic animals [4]–[16], [19], [20] transgene expression in spermatozoa was reported. Spermatozoa are highly specialized germ cells, which have to actively roam through uterus and oviduct to fertilize an oocyte. Whether the incorporation of Venus fluorophores into boar spermatozoa is compatible with a functional status of these cells was not known.

Venus is a yellow shifted variant (excitation maximum at 515 nm) of the commonly used enhanced green fluorescent protein (EGFP, excitation maximum at 488 nm). Both fluorophores extend over 239 amino acids and share an amino acid-identity of 97% [30]. The fluorescence originates from an internal amino acid sequence, which is post-translationally modified to form an imidazolidone ring. Specific fluorophores, but also EGFP, are thought to produce oxygen radicals and might act as light-induced electron donors in photochemical reactions with biologically relevant electron acceptors [30], [31]. These effects might contribute to the toxicity of fluorophore proteins observed in some studies [32]–[34]. The vast number of viable transgenic animals with expression of EGFP or other fluorophores, however, argues against a gross toxicity of these proteins during ontogenesis [35]–[37]. Fluorophore-loaded spermatozoa could be a sensitive cell system for the assessment of subtle effects of marker expression. Mature spermatozoa are motile primary cells, which can be isolated in a fully functional status under defined conditions, and morphological, biochemical and biophysical criteria are well defined for determination of sperm quality [38].

Here, we characterized the prominent Venus expression in spermatozoa of transposon-transgenic boars [17], [18], assessed, whether the Venus fluorescence reflected active transcription in boar sperm cells, and determined whether the high expression of an ectopic fluorescent protein affected reproductive parameters in transgenic pigs. In addition, the relationship between phenotype and genotype of male germ cells from transposon-transgenic boars with regard to the Venus trait was analyzed.

## Results

### Fluorescence microscopy and flow cytometric measurements of transgenic spermatozoa

**Figure 1:**
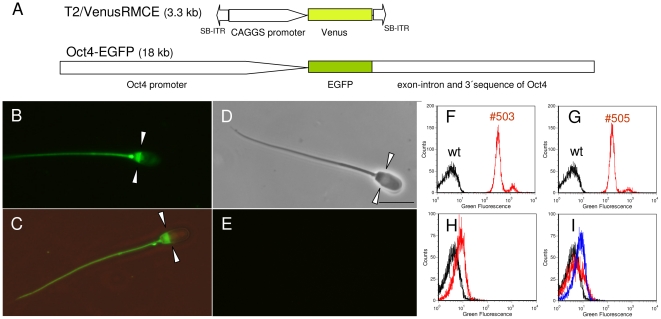
Gene constructs and Venus-expression in porcine spermatozoa. A) Schematic depiction of fluorophore constructs. The Venus transposon (T2/VenusRMCE) consists of a *CAGGS* promoter and *Venus*-cDNA flanked by *Sleeping Beauty* inverted terminal repeats (SB-ITR). The *Oct4-EGFP* carries 9 kb Oct4 promoter, followed by *EGFP*-cDNA and 9 kb of *Oct4* exon-intron sequences. Drawing is not at scale. B) Representative Venus fluorophore expression in spermatozoa of transposon transgenic boar #505 viewed under specific excitation (3 ejaculates were analyzed); C) Venus expression in spermatozoa (#503) viewed under specific excitation and dim bright light (3 ejaculates were analyzed). Note the differential Venus fluorescence in subacrosomal sheath and PAS. Arrow heads indicate the equatorial rim. D), E) Spermatozoa from a wildtype boar under bright light and specific excitation (bar  =  10 micrometer); F), G) Flow cytometric measurements of Venus-fluorescence in sperm of transposon transgenic boars #503 and #505 (red lines, n = 6)) and a non-transgenic control sample (black). Mean fluorescence intensities: wt  =  3.3; #503  =  350 ; #505  =  147. The small peaks (red lines) represent somatic cells. H), I) Flow cytometric measurement of specific EGFP fluorescence in sperm from three individual Oct4-EGFP transgenic boars (red and blue lines) and a wildtype control (black). Mean fluorescence intensities: wt  =  3.4; #252  =  7.6; #265  =  4.6; #255  =  7.6.

Two transposon transgenic boars [17], [18], each carrying three monomeric integrants of an ubiquitously active CMV early enhancer, chicken beta actin (CAGGS) promoter driven Venus construct (Fig. 1A) and three cloned transgenic boars (F0), carrying an Oct4-EGFP construct [39], [40], were analysed (Table 1). The transcriptional activity of the Oct4 promoter is restricted to the germline, and EGFP fluorescence was exclusively detected in blastomeres of cleavage-stage embryos, germline cells of genital ridge and testis [39], however, ejaculated spermatozoa have not been analysed before.

**Table 1:** Transgenic boars used for spermatozoa analysis.

Animal ID	Transgene	Generation	Expression pattern	Transgene copy no.	Transgenesis method	Ref.
#503	CAGGS-Venus transposon	F0	ubiquitous	3	CPI	[17], [18]
#505	CAGGS-Venus transposon	F0	ubiquitous	3	CPI	[17], [18]
#252	Oct4-EGFP	F0	germ line specific	3	SCNT	[39]
#255	Oct4-EGFP	F0	germ line specific	3	SCNT	[39]
#265	Oct4-EGFP	F0	germ line specific	2	SCNT	[39]

CPI, cytoplasmic plasmid injection into zygote; SCNT, somatic cell nuclear transfer.

Amazingly, a distinct compartmentalization of Venus-fluorescence was apparent in spermatozoa of both transposon-transgenic boars (Fig. 1B-E, Video S1). Spermatozoa of boars #503 and #505 showed prominent Venus-fluorescence in the postacrosomal sheath (PAS), just below the equatorial rim, as well as in the midpiece and the tail (Fig. 1B, C). The tail tips of spermatozoa from both founders were negative or significantly less fluorescent. Flow cytometric measurements confirmed prominent Venus expression in sperm of transposon-transgenic boars (Fig. 1F,G). High resolution confocal microscopic analysis of spermatozoa from transposon-transgenic boar #505 is shown in Fig.S1. All spermatozoa of the transposon-transgenic boars were Venus-positive and no difference in fluorescence intensity was found between spermatozoa. This finding raised the question, whether the CAGGS-promoter drives transcription in mature spermatozoa, or whether Venus-transcripts persisted in porcine spermatozoa. No specific EGFP signal could be detected in semen samples from three cloned boars carrying an Oct4-EGFP transgene by fluorescence microscopy (data not shown); and no or only minimal specific EGFP fluorescence was detected by flow cytometry (Fig.1H, I).

### RNA extraction and measurement of Venus transcripts in spermatozoa

**Figure 2:**
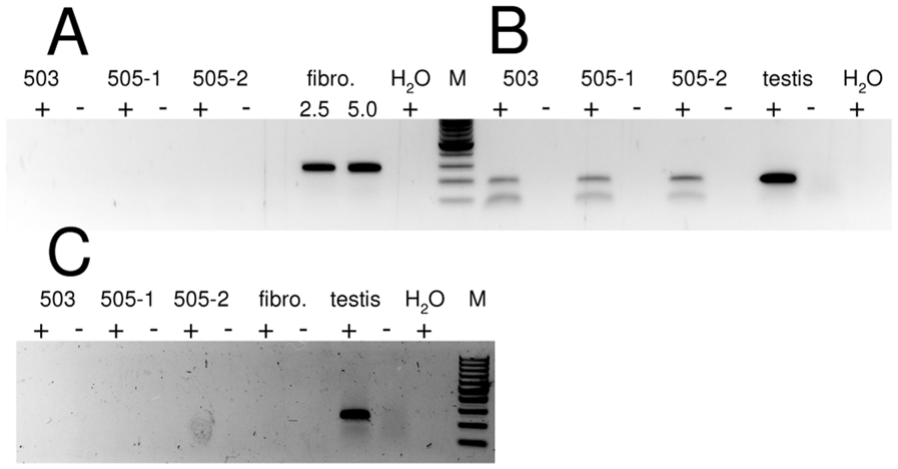
Absence of Venus transcripts in spermatozoa-specific RNA. A) Absence of *Venus* mRNA in boar spermatozoa; *Venus*-specific RT-PCR from the indicated sperm samples of boars #503 and #505, fibroblast (#505) and no template. For fibroblasts (#505) samples 2.5 and 5 ng of cDNAs, for the spermatozoa samples 60 ng of cDNAs were used as templates. From #505 ejaculates, two independent RNA-isolations were assayed. B) The sperm-specific *Protamine 1* transcripts are detectable in all isolations of sperm-specific RNA: *protamine1* (*PRM1*)-specific RT-PCR from the indicated sperm samples of boars #503 and #505, and testis (wt). +, with RT; - without RT; H_2_O, no template. From #505 ejaculates, two independent RNA-isolations were assayed. C) The hematopoietic-specific marker *CD45* is expressed only in testis, but not in fibroblasts or Percoll purified spermatozoa. 503, 505-1, 505-2: indicate different RNA-preparations of spermatozoa from transposon-transgenic boars; fibro, testis indicate RNA preparations from transgenic fibroblasts and wildtype testis; +, - indicate the addition, or omission of RT; H_2_O is the no template control.

To determine whether the Venus fluorescence reflected active transcription or presence of paternally inherited transcripts in the spermatozoa, ejaculated sperm from the transposon-transgenic boars was centrifuged over a 90% Percoll gradient to remove leukocytes and epithelial cells (Fig. 2; Fig.S2). Total RNA was then extracted from purified sperm fractions. The presence of the protamine 1 RNA (PRM1), which is a typical RNA of sperm, and the absence of the hematopoietic cell marker CD45 by RT-PCR suggested successful isolation of sperm-specific RNA (Fig. 2). A specific RT-PCR for the Venus sequence reproducibly failed to amplify an amplicon from sperm RNA samples, suggesting the absence of Venus transcripts in mature porcine spermatozoa.

In addition, a Western blot analysis confirmed presence of the fluorophore protein in Percoll purified spermatozoa from transposon-transgenic boars (Fig.S3). The ubiquitous expression of Venus in testis tissues was documented by histological analyses of neonatal and adult tissue samples (Fig.S4).

### Fertility assessment of Venus fluorophore-positive spermatozoa

To determine whether the Venus fluorescence in spermatozoa has an influence on morphology and motility of the sperm cells, essential sperm parameters of ejaculates from both transposon-transgenic boars (#503 and #505) were analysed and compared with that of wildtype controls (Table 2). While semen of boar #503 did not show any essential differences compared with the controls, spermatozoa of boar #505 showed a modestly reduced straight-line-velocity and linearity than the controls. The examination results suggested that ejaculates from the transposon-transgenic boars fulfilled standard semen quality requirements.

**Table 2:** Semen parameters measured in fresh samples from transgenic and control boars.

Semen parameter	#503 (mean ± SD)	#505 (mean ± SD)	Control wildtype (mean ± SD)
Motility (MOT) (%)	76±8	75±4	83±5
Progressive motility (PMOT) (%)	45±20	41±4	54±9
Velocity straight line (VSL) (µm/s)	30±8^ab^	25±2^b^	34±4^a^
Linearity (LIN) (%)	22±2^a^	17±0.7^b^	23±2^ac^
Morphological defects (%)	9±4^a^	11±3^a^	22±12^b^

n = 4 replicates.

a-c Different letters indicate significant differences (P<0.05) between boars.

### Venus fluorescence distribution among X and Y chromosome-bearing spermatozoa

**Figure 3:**
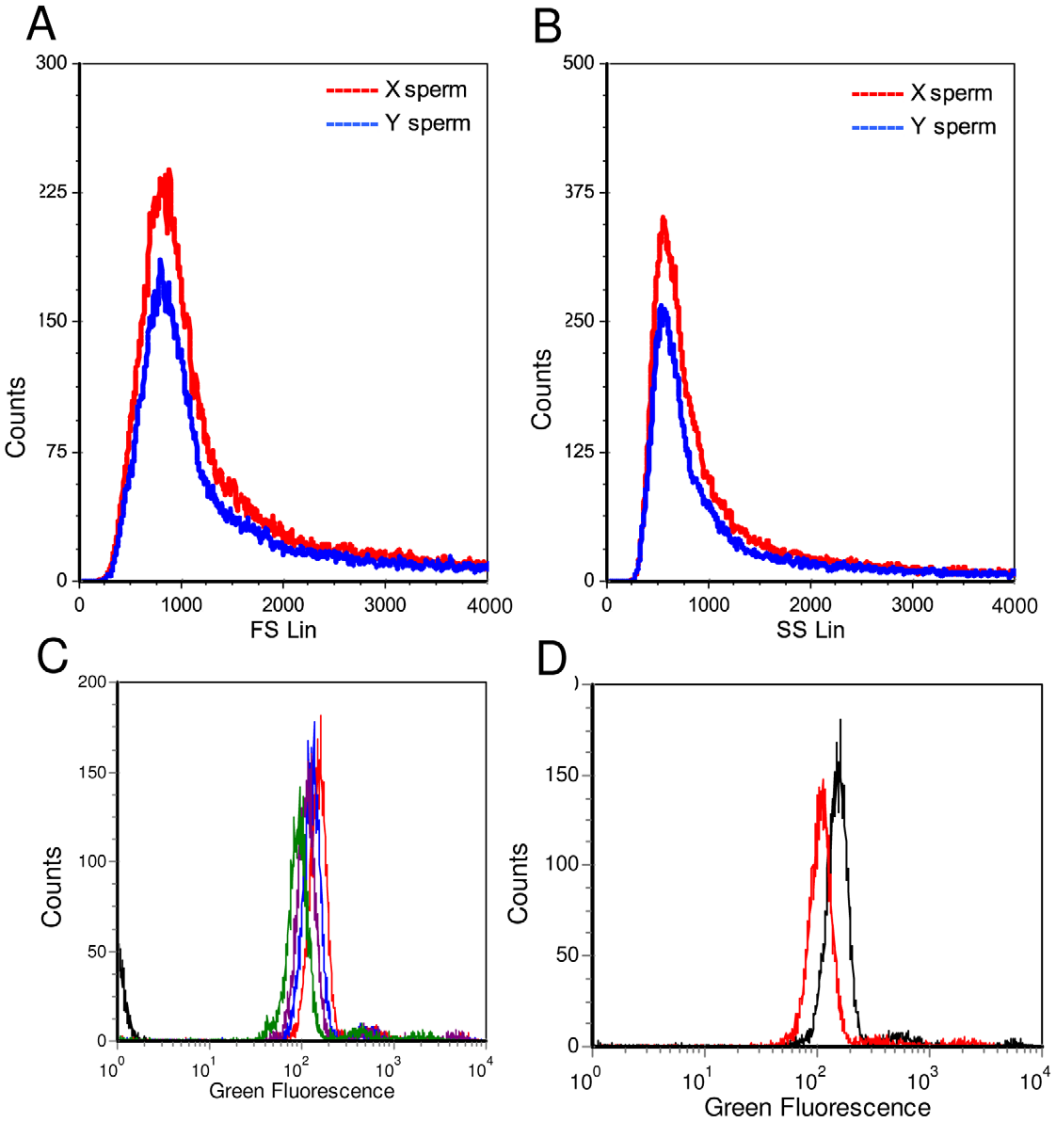
Uniform Venus fluorescence of X and Y chromosome bearing spermatozoa. A), B) Venus fluorescence (forward and side signal) of oriented sperm from boar #503, after flow cytometric separation in X and Y chromosome bearing populations. C) Flow cytometric measurement of Venus-fluorescence in sperm of transposon transgenic boar #503 at four time points (0 min (red line, 30 min (blue line), 60 min (lilac line), 180 min (green line) of exposure to blue LED light, and a non-transgenic control boar sample (black line on the very left). Mean fluorescence intensities: Wt = 1. #503 (0 min = 153, 30 min = 130, 60 min = 113, 180 min = 93). D) Decrease of Venus fluorescence intensity in whole semen after long-term storage. Venus fluorescence was assessed directly after isolation (black line) and after storage at 4°C for four weeks (red line).

Spermatozoa from the transposon-transgenic boars were sorted according to the sex chromosomes (flow sorter MoFlo, Beckman-Coulter) and subsequently Venus fluorescence signals were determined for X and Y chromosome-bearing populations. No difference was found for Venus signals among X and Y chromosome-bearing sperm populations neither for forward nor for side fluorescence (Fig. 3A, B), albeit the size difference of the sex chromosomes results in a relative disparity of ∼ 3% of total DNA between these spermatozoa fractions [38]. Thus, this analysis corroborated uniformity of spermatozoa with respect to Venus fluorescence.

### Blue light excitation of Venus-loaded spermatozoa

To assess whether specific light-induced electron donation of the Venus fluorophore might be detrimental for spermatozoa, sperm from transposon-transgenic boars and control samples were placed under a high power blue LED (40 W) to specifically excite Venus molecules. Membrane integrity and motility of spermatozoa were recorded over a time course of three hours. During exposure to blue LED light the membrane damage did not increase significantly, compared to control samples (Table 3). To determine whether exposure to blue LED light has an influence on morphology and motility of sperm, ejaculates from both transgenic boars (#503 and #505) and controls were analysed for a range of routinely applied fertility parameters (Table 4). After 3 hours of exposure, motility of spermatozoa from transgenic and non-transgenic boars decreased to nearly zero, suggesting that blue light excitation is detrimental for sperm motility independent of Venus expression. During extended exposure periods of blue LED excitation, the fluorescence intensity of transgenic sperm decreased slightly (Fig. 3C). To assess stability of Venus protein in boar spermatozoa, the transgenic sperm probes were stored at 4°C in the dark for up to five weeks. Spermatozoa from boars #503 and #505 showed a 10–20% decrease of fluorescence intensity after long-term preservation (Fig. 3D).

**Table 3:** Effects of Venus excitation on membrane integrity of spermatozoa.

Animal ID	membrane intact sperm (%)				Untreated controls
	after blue LED exposure for				
	0 min	30 min	60 min	180 min	180 min
#503	93.3	94.4	91.5	89.9	93.3
#505	88.2	87.5	88.8	76.9	85.4
Wt (n = 2)	92.0-95.8	92.8	89.8-92.5	86.7-88.4%	91.2-93.8

n = 3 replicates.

**Table 4:** Effects of Venus excitation on sperm motility.

Animal ID	CASA	Ratio of motile spermatozoa (%)				Untreated controls
		after blue LED exposure for				
		0 min	30 min	60 min	180 min	180 min
boar # 503	MOT %	75	71	36	8	79
	PMOT%	36	36	10	2	24
boar # 505	MOT %	69	71	51	3	56
	PMOT%	44	46	27	0	32
wildtype	MOT %	70	45	17	2	54
	PMOT%	41	24	6	0	22

n = 3 replicates.

### Fate of Venus protein in early embryos, inheritance of transgene and offspring analysis

**Figure 4:**
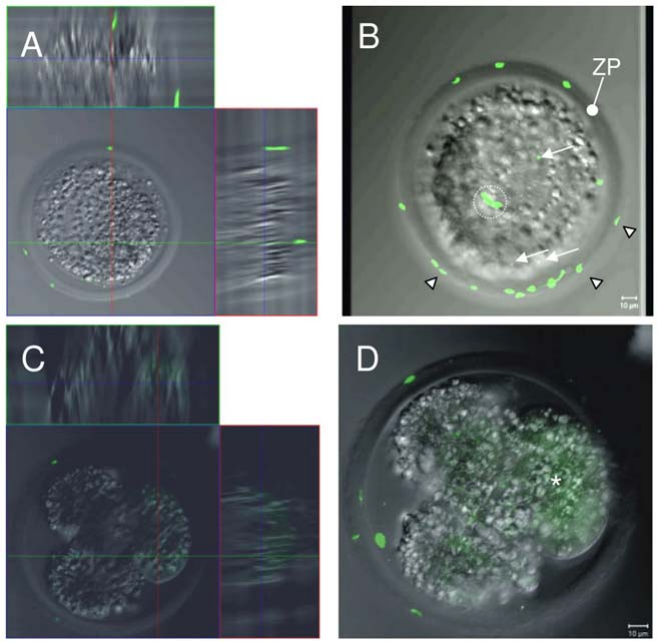
Venus fluorophore transmission to zygotes. Venus fluorescence was analysed in fertilized zygotes to 4-cell stages by confocal microscopy, and overlays of DIC and Venus channels are shown. A): Single optical section of 2 micrometer thickness from the equatorial region of a zygote, together with the orthogonal xz and yz views. B) Corresponding projection of a series of optical planes. This zygote contains three discrete Venus fluorescent spots inside the cytoplasm (arrows), but not in the pronuclei. Most likely, these reflect remnants of postacrosomal sheath and mid piece of one spermatozoon. Note, the intensive fluorescence of spermatozoa at the outside of the zygote, attached to the *Zona pellucida* (ZP) (some are marked with white triangle). Due to the projection, spermatozoa, which are attached to the top or the bottom appear seemingly to be inside the zygote (dashed circle). However, an analysis of the individual optical sections allows an unequivocal determination of their position. C) Single optical section of 2 micrometer thickness of a 4-cell stage, together with the orthogonal xz and yz views. D) Corresponding projection of optical planes. Asterisk indicates blastomere with de novo synthesized Venus protein.

To analyze fate of the Venus protein after fertilization, two wild-type sows were inseminated with semen of the transgenic boars, embryos were flushed from oviduct, and early embryos were analyzed by confocal microscopy. All analyzed zygotes (n = 6), 2-cell stage embryos (n = 4) and 4-cell stage embryos (n = 5) contained discrete spots of Venus fluorescent material within their cytoplasm (Fig. 4). This material most likely represents remnants of the spermatogonial theca and of the mid piece. In addition, several spermatozoa bound to the *Zona pellucida*, a glycoprotein membrane covering the embryo, were detected by their Venus fluorescence. Transcriptional activation of the porcine genome occurs at the 4-cell stage [41], and individual blastomeres were found to show de novo expression of the transgene.

**Figure 5:**
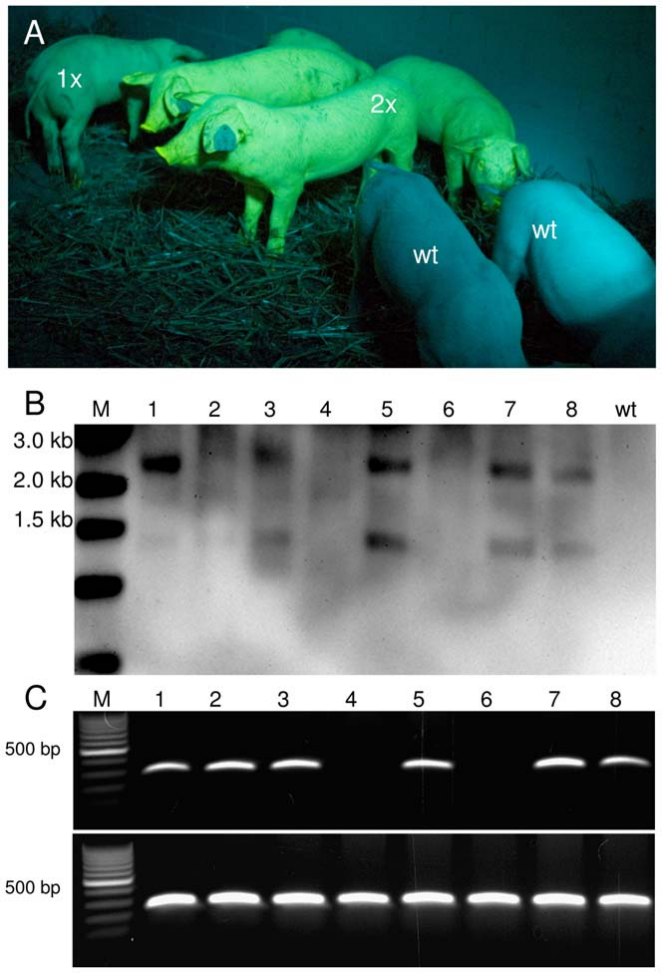
Segregation of Venus transposon in F1 offspring. A) F1-offspring viewed under specific excitation of Venus. Under the recording conditions, Venus fluorescence is displayed as green-yellow color, exemplarily some animals are labelled with their copy number of the transposon. Note that the copy numbers of the transposon correlated directly with the fluorescence intensity. The non-transgenic littermates (wt) appear bluish due to reflected and scattered excitation light B) Southern blot analysis of one litter. Lanes 1-3, 5, 7 and 8 represent transposon transgenic piglets with specific hybridization signals. Lanes 4 and 6 represent non-transgenic littermates. M, molecular size marker. C) PCR genotyping for the presence of the Venus transgene. As positive control, a PCR for polyA polymerase (PolyA) was included; wt, wild type sample.

To analyze inheritance of the Venus transgene copies, ejaculated sperm of the transposon-transgenic boars was used to inseminate 8 sows, of which 7 became pregnant. Four pregnant sows were sacrificed at day 30 post insemination and a total of 42 fetuses were recovered. 36 fetuses were Venus-fluorescent and 4 fetuses did not show specific fluorescence. Phenotypically, graded fluorescence intensities were obvious between the marker positive fetuses. This phenotypic characterization was subsequently confirmed by PCR analysis and Southern blotting of genomic DNA. A direct correlation between copy number of the Venus transgene and the fluorescence intensity was found. In addition, Southern blotting proved segregation of the independent transposon integrants (Table 5). Three sows, which went to term, delivered a total of 32 piglets and two mummies. 29 of the piglets were Venus-transgenic and expressed the transgene, and 3 piglets were non-transgenic (Table 5, Fig. 5). Corroborating the fetal data, different grades of fluorescence intensity between littermates were evident, and could be positively correlated with the transgene copy number of the respective animals. The molecular analysis of the F1-offspring indicated independent segregation of transposons. Approximately 10% of the 74 F1-offspring were non-transgenic (Table 5).

**Table 5:** Phenotypes and genotypes of F1-offspring.

Parental boar ID	Status of F1 offspring	No. of offspring	No. of Venus expressing offspring, (%)#	No. of non-fluorescent offspring§, (%)
#503	fetus (day 30 p.c.)	21	17 *, (81.0)	2, (9.5)
#505	fetus (day 30 p.c.)	21	19, (90.5)	2, (9.5)
#503	piglets	32	29, (90.6)	3, (9.4)

*2 degraded fetuses could not be analyzed,

# with 1-3 transposon integrants;

§ genotypic wild type.

## Discussion

Here, we report boars transgenic with CAGGS-Venus transposons showing a unique phenotype of fluorescence marker load in mature spermatozoa, in addition to expression in most other cell types. The transgenic boars were generated via CPI of *Sleeping Beauty* vectors into porcine zygotes [17], [18]. In mammalian spermatozoa the sperm nucleus is surrounded by a condensed cytosolic layer, the perinuclear theca, which is divided in subacrosomal layer and postacrosomal sheath (PAS) [42], [43]. Essentially no Venus fluorescence was found in the subacrosomal layer of spermatozoa from the transposon-transgenic boars, but a prominent localization of Venus was evident in the PAS, below the equatorial segment region. In addition, Venus fluorescence was prominent in midpiece and in tail. Spermatogenesis is a complex process, where spermatids undergo dramatic remodelling, condensation of nuclear chromatin, biogenesis of perinuclear theca, formation of flagellum and development of acrosome to form functional spermatozoa [42], [43]. In bovine spermatozoa it has been postulated that the PAS is assembled during sperm elongation, and contains histone proteins and a presumptive oocyte-activating factor dubbed PAWP (resides in the PAS and contains a consensus binding site for group I WW domain proteins) [43]. Mature spermatozoa have lost most of their cytoplasm, and have been commonly thought to be transcriptionally and translationally dormant [44], [45]. Nevertheless, the presence of remnant mRNAs has been shown in mature spermatozoa of several mammals [42], [44]–[47].

The prominent expression of a reporter fluorophore in boar spermatozoa raised the questions, whether this is a species-specific phenomenon or due to the used transposon-construct, and whether the fluorescence correlates with presence of Venus transcripts and potentially of active translation in mature boar spermatozoa. No or only minimal specific fluorescence was found in spermatozoa from three boars transgenic carrying a germline restricted Oct4-EGFP construct, albeit expression of EGFP in most germline cells and prospermatogonia was shown [39]. Similarly, transgenic boars, produced by lentiviral transfection of an ubiquitously active phosphoglycerate kinase promoter-EGFP construct did show only minimal specific fluorescence in mature spermatozoa [48]. Thus, prominent transgenic expression in porcine spermatozoa is not a general phenomenon in this species.

Until recently, it was thought that spermatozoa do not contain RNA, but detailed analysis revealed that certain transcripts [44], such as PRM1 mRNA, are present in sperm [44]-[47], [49], [50]. In case of the Venus-transposon transgenic boars a highly sensitive RT-PCR analysis suggested that the spermatozoa did not contain Venus transcripts. Thus, Venus-fluorescence in spermatozoa seemed to be due to Venus protein already translated in prospermatogonial stages. An equal distribution of Venus protein to all spermatozoa most likely occurred via cytoplasmic bridges, connecting immature spermatids at the syncytial stage [51]–[53].

**Figure 6:**
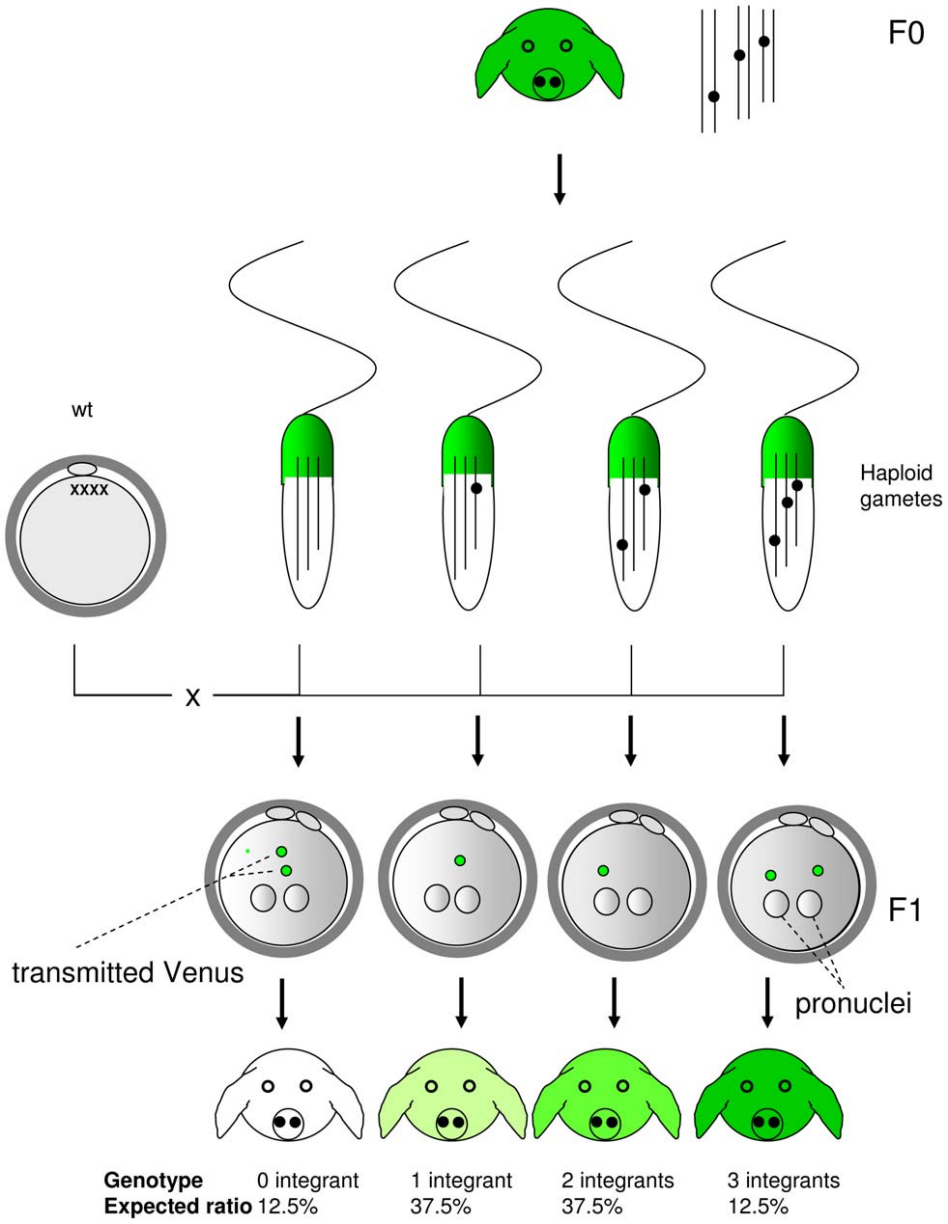
Genotype-independent transmission of a transgenic fluorophore protein by spermatozoa. In founder (top) and F1-offspring (bottom) a direct correlation between genotype and phenotype is found. The genotype is depicted by lines representing chromosomes, dots indicate monomeric copies of the transgene; the phenotype is depicted by graded intensities of green color. However, in mature haploid spermatozoa a uniform phenotype is found, despite segregation of transgenes and a different transgene load in different sperm cells. Even the ∼ 10% spermatozoa, which did not carry any transgene copy, showed a uniform Venus expression. The data presented here, suggest that the uniform phenotype in spermatozoa is due to a highly accurate distribution of Venus protein between immature spermatozoa. Most likely several paternal proteins might be equally distributed by this mechanism between immature spermatozoa.

Both Venus-transposon transgenic boars carried three monomeric integrations of the transgene, which segregated during meiosis. Whereas all spermatozoa showed a uniform phenotype with regard to Venus fluorescence, the transgenic trait segregated in the offspring (Fig. 5). Assuming three independent integrations and a Mendelian inheritance, 12.5% of offspring should carry 3 transposon-integrations, 37.5% should carry 2 integrations, 37.5% should carry 1 integration and 12.5% should be non-transgenic (Fig. 6). Indeed, approximately 10% of the offspring were found to be genotypic (and phenotypic) non-transgenic. These findings strongly support the notion that mature spermatozoa displayed a phenotype, which differed from their haploid genotype. The carry-over of the Venus fluorophore in a genotype-independent manner to an oocyte, represents an example for a non-genetic contribution to a mammalian embryo [54], [55]. Most likely the “epigenetic” contribution of Venus protein will only be transiently effective after fertilization. Thus, the uniformity in spermatozoa with regard to Venus-specific fluorescence did not reflect the genotype, but likely the distribution of cytoplasmic components via syncytial bridges during spermiogenesis. Data presented here support the assumption of a non-genetic transmission of a fluorophore protein thereby extending the knowledge on spermatozoa-transmitted epigenetic nuclear factors, such as miRNAs, RNAs and DNA modifications [54]–[56]. Due to extreme reduction of the cytoplasm in mature spermatozoa, transmission of cytoplasmic proteins is not well studied, except for factors responsible for distortion of sex transmission ratios [57] and factors which may be of importance for fertilization [43].

The molecular basis for enrichment of Venus fluorophores in PAS, midpiece and tail, and the apparent absence in the subacrosomal layer is not known at the moment. However, the uniform distribution of the Venus reporter in all spermatozoa suggests that a specific mechanism is responsible for this process, and most likely organizes the equal distribution of several paternal proteins in immature spermatids, extending previous observations of an extensive cellular traffic in spermatids of the rat [53]. Even sophisticated analysis by sperm sorting according to X and Y chromosome bearing spermatozoa fractions did not reveal any differences in Venus fluorescence in sperm from transposon-transgenic boars. The potential effects of spermatozoa-mediated non-genetic transmission of proteins on fertilized zygotes is not assessable by commonly applied techniques like in situ hybridization, RT-PCR or DNA array analysis of early embryos [52], [58] and might require sophisticated proteomic and live imaging techniques.

Apparently, the Venus fluorophore load in boar spermatozoa behave neutral and did not affect fertilization capacity, vitality of embryos or litter size. The born F1 piglets are vital, develop normally and except the transgenic trait no difference between transgenic piglets and their non-transgenic littermates was found. This observation extends previous assessments of reproduction performance of boars transgenic with a cytomegalovirus promoter (CMV) driven alpha-1,2fucosyltransferase gene [59], [60], albeit it is not clear whether the CMV promoter is active in porcine spermatogonial cells.

The data presented here, extend the knowledge about distribution mechanisms of paternally derived proteins in pre-spermatogonia and might be stimulating for research in non-genetic transmission by spermatozoa. It is tempting to speculate that transmitted protein components may affect early developmental processes in the embryo.

## Materials and Methods

### Ethics statement

Animals were maintained and handled according to German animal welfare guidelines and German GMO regulations. The animal experiments were approved by an external ethics committee (Niedersächsisches Landesamt für Verbraucherschutz und Lebensmittelsicherheit, Oldenburg, Germany, AZ 33.9-42502-04-09/1718).

### Collection of ejaculated sperm, artificial insemination, recovery of embryos

Gilts were superovulated by i.m. injection of 1.000 IU Intergonan/PMSG (96h before insemination) and 500 IU Ovogest/hCG (24 h before insemination) and then artificially inseminated on day 0 (d0). In some cases, pregnant recipients were sacrificed at days 1 and 2 post inseminations and embryonic stages were isolated by flushing excised oviducts with PBS, supplemented with 1% newborn calve serum.

Sperm-rich fractions were collected from boars using a dummy and by gloved – hand technique. Semen samples were extended with Androhep (1∶2) and transported to the laboratory at 37°C. Sperm concentration was determined by NucleoCounter SP-100 system (ChemoMetec, Denmark). For experiments the sperm concentration was calibrated to 10^8^/ml. Sperm analysis included motility, morphology and membrane integrity.

### Fluorescent microscopy and macroscopic excitation of Venus fluorophore

For fluorescence microscopy, images were obtained with an Olympus BX60 (Olympus, Hamburg, Germany) fluorescence microscope equipped with a 12-bit digital camera and Cell*P software (Olympus DP 71). For Venus fluorescence an excitation filter of 460–490 nm, a band pass emission filter of 515–550 nm and a dichroic mirror DM505 (Olympus) were used. Video sequences of spermatozoa were filmed with the AVI recorder function of Cell*P. For specific excitation of live Venus-transgenic piglets and pigs, a blue floodlight LED (40 W; euroliteGermany, Germany) and an electronic camera (Canon Powershot) equipped with a yellow emission filter was used.

### Confocal microscopy

Unfixed porcine zygotes, 2-cell and 4- cell embryos were embedded in Vectashield (H-100, Axxora, Lörrach, Germany) and sealed with a coverslip. Venus-specific fluorescence was investigated with a confocal laser scanning system LSM510 (Carl Zeiss, MicroImaging GmbH, Jena, Germany) connected to an Axioplan 200 (Carl Zeiss). For excitation an argon laser (514 nm), and for detection a band pass of 530–560 nm were used. The gross cell morphology was visualized in multitracking mode with the transmisssion channel in differential contrast (DIC). All images were taken with a 20x Plan-Apochromat, na. 0.75 (Carl Zeiss). Control embryos resulting from inseminations with semen from wild type boars (n = 2) did not reveal any signal in the Venus specific fluorescence channel.

### Histology

Unfixed tissues were viewed in a stereomicroscope (Olympus SZX16) equipped with 0.5x and 1.6x planapo objectives and fluorescence optics. For histological examinations, tissue samples were fixed in 4% formaldehyde overnight, soaked in a 20% sucrose solution for 24 hours, and frozen in embedding medium (MICROM Laborgeräte, Walldorf, Germany) and 10 micrometer sections were cutted on a MICROM cryomicrotome (ThermoFisher, Dreieich, Germany), embedded in Vectashield and viewed in an Olympus BX60 fluorescence microscope.

### Flow cytometry

Flow cytometry analysis of primary cells, leukocytes and spermatozoa was performed using a FACScan (BD Bioscience, Heidelberg, Germany) equipped with an argon laser (488 nm, 15 mW). Samples were diluted to 0.5×10^6^ cells/ml and measured in duplicates acquiring 10 000 cells per sample. Membrane impaired cells were excluded from analysis by adding propidium iodide to a final concentration of 20 µM.

### Sex chromosome sorting of spermatozoa

To determine, whether differences in Venus fluorescence exist between X and Y chromosome bearing spermatozoa, ejaculates were collected and diluted with sample fluid (modified Androhep) to 10^8^sperm/ml. Samples were sorted according to the Beltsville Sperm Sexing method. Briefly, sperm were labelled with 20 µl of an 8.9 mM Hoechst 33342 solution and were incubated at 34 °C for 75 minutes. Labelled and incubated spermatozoa were kept at 22 °C in the dark. Aliqouts of 1 ml were sorted in a flow sorter (MoFlo, Beckman-Coulter, FL, USA) using PBS supplemented with 1% BSA as sheath fluid and a solid state UV Laser (Coherent, Dieburg, Germany) working at 200 mW. The optical system of the sorter was modified, using a PMT (FL1) instead of a forward scatter diode. Signals were triggered on the side fluorescence of the sperm heads recognized by a 90° PMT. All sorted cells were collected in TEST-yolk [61] containing 2% seminal plasma. After collection samples were centrifuged at 840 g for 20 minutes. The supernatant was removed and the sperm pellet was re-suspended with Androhep and batches were pooled to give a sperm concentration of 10^8^ sperm/ml.

One millilitre of the sex selected sperm populations were passed through a flow sorter again. The sorter was equipped with a 488 nm solid state laser (iCyte, Ill, USA) set to 35 mW to measure the amount of Venus fluorescence. An aliquot of the ejaculate served as control and was treated identically, except that it was not labelled with Hoechst 33342 and was not passed through the sorter.

### RNA isolation from porcine sperm

Somatic cells were removed from ejaculated sperm by Percoll gradient (90%) centrifugation for one hour. The sperm pellet was extracted by TriReagent for 30 minutes, centrifuged (12′000 g) and the supernatant suspended in chloroform. After 12′000 g centrifugation, 400 microliter of supernatant were combined with 1 microliter glycogen (5 mg/ml) and 400 microliter 2-propanol. After centrifugation at 12′000 g for 15 minutes, the pellet was washed with 70% ethanol, dried and resuspended in 10 microliter pure water. To remove any contaminating DNA, a DNA-digest was performed with DNase (Epicentre), before reverse transcription and PCR detection. The primers and PCR conditions are given in Table S1.

### CASA measurements

The motility parameters of the spermatozoa were measured with a computer-assisted sperm analyser (CASA; Hamilton Thorne Bioscience-IVOS, Beverly, USA) using a Makler chamber. The motility parameters measured were as follows: MOT% (percentage of total motile sperm), PMOT% (percentage of progressive motile sperm), VSL (progressive velocity, µm/s), and LIN (linearity of track %, VSL/VCL). To analyse the morphology of sperm, 20 µl of sample (100 Million per ml) were added to 200 µl Hancock's solution (4% formol citrat). A total of 200 spermatozoa were counted using a phase contrast microscope at 1000 x magnification and using the oil immersion technique. Each sperm was evaluated for acrosome integrity, as well as total morphologic integrity.

### Genotyping of offspring

In brief, genomic DNA was isolated with the proteinase K method. PCR genotyping was performed as described [18]. For Southern blotting genomic DNA was digested with NcoI, separated on a 0.6% agarose gel by electrophoresis and blotted on a PVDF membrane. Then a transgene specific probe labelled with digoxigenin was used for hybridization as described [18].

### Western blotting

Western blotting was done as described [62]. In brief, spermatozoa were extracted in RIPA buffer and 10 microgram of protein per slot was separated on a 12% SDS-PAGE gel, blotted to a PVDF membrane, blocked in 5% non-fat milk powder and probed with a rabbit polyclonal anti-EGFP antibody (Thermo Scientific, 1∶1000), followed by a secondary antibody (anti-rabbit IgG-horseradish peroxidase; 1∶10 000, Sigma). In addition, a murine monoclonal anti-tubulin antibody (Developmental Hybridoma Bank, E7, 1∶1000) and a anti mouse IgG-horseradish peroxidase antibody (Sigma, 1∶20 000) were employed. For detection an ECL+ kit (GE Healthcare) and an image acquisition system (Vilber Lourmat, Fusion SL3500) were used. A MagicMark protein size ladder (Invitrogen) was used.

## Supporting Information

Figure S1 **Compartimentalized Venus localization in spermatozoa.** Confocal microscopic images of spermatozoa from boar #505 are shown in A) differential interference contrast (DIC), B) DIC and Venus fluorescence, and C) in Venus-specific channel. Apparently, the distribution of Venus protein is dynamic, sperm cells marked with an asterisk represent “fresh” sperm, whereas after short incubation times (or handling procedures) a relocation into the postacrosomal sheath happens (arrow). Bar  =  5 micrometer.
(TIF)

Figure S2 **Percoll purification of sperm cells.** A) Low magnification view of unpurified Venus-positive spermatozoa. Note the aggregates of somatic cells (epithelial and immune cells), some are labelled by arrows. B) Percoll purified Venus-positive spermatozoa, and C) Brightfield view of B).
(TIF)

Figure S3 **Detection of Venus protein in sperm of transgenic boars.** Western blot detection of Venus protein (molecular weight ∼ 30 kilodalton (kD)). Loading of slots: M, molecular size ladder (bands of 20, 30, 40 and 50 kD are indicated); 1-4 protein extracts isolated from: 1, wild type sperm; 2, wild type sperm, Percoll purified; 3, sperm from transgenic boar, 4, sperm from transgenic boar after Percoll purification.
(TIF)

Figure S4 **Expression of Venus in transgenic testis.** A) Specific Venus fluorescence in testis and accessory glands of a transgenic F1 piglet (day 7 postpartum), which succumbed to a bacterial infection, is shown. Inset, same view under brightfield conditions. B) Venus fluorescence in adult testis (18 months F0 boar). Bars  =  2.5 cm. C) Venus fluorescence in cryosection of boar testis and D) corresponding brighfield view.
(TIF)

Table S1 **Primer pairs used for RT-PCR.**
(DOC)

Video S1 **Venus fluorescent spermatozoa.** Freshly isolated spermatozoa from boar #505. The start sequence shows the spermatozoa under brightfield conditions, followed by specific excitation of the Venus fluorophore. Note, several spermatozoa are attracted to the border of an air bubble in the lower halve of the display window.
(WMV)
