# Supplementary figures and images for: Genotype-Independent Transmission of Transgenic Fluorophore Protein by Boar Spermatozoa
Source: PLoS One. 2011 Nov 16;6(11):e27563. doi: 10.1371/journal.pone.0027563 (PMC3217978; doi:10.1371/journal.pone.0027563)

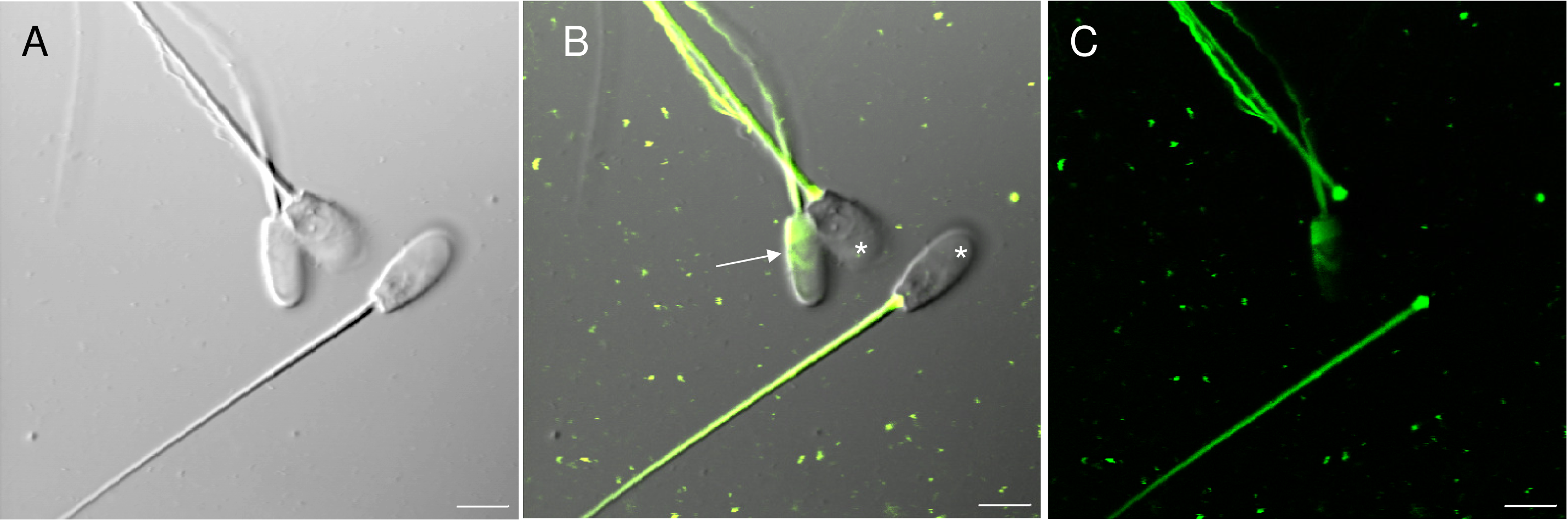

Supplement: Figure S1 — Compartimentalized Venus localization in spermatozoa. Confocal microscopic images of spermatozoa from boar #505 are shown in A) differential interference contrast (DIC), B) DIC and Venus fluorescence, and C) in Venus-specific channel. Apparently, the distribution of Venus protein is dynamic, sperm cells marked with an asterisk represent “fresh” sperm, whereas after short incubation times (or handling procedures) a relocation into the postacrosomal sheath happens (arrow). Bar = 5 micrometer. (TIF) [file pone.0027563.s001.tif]

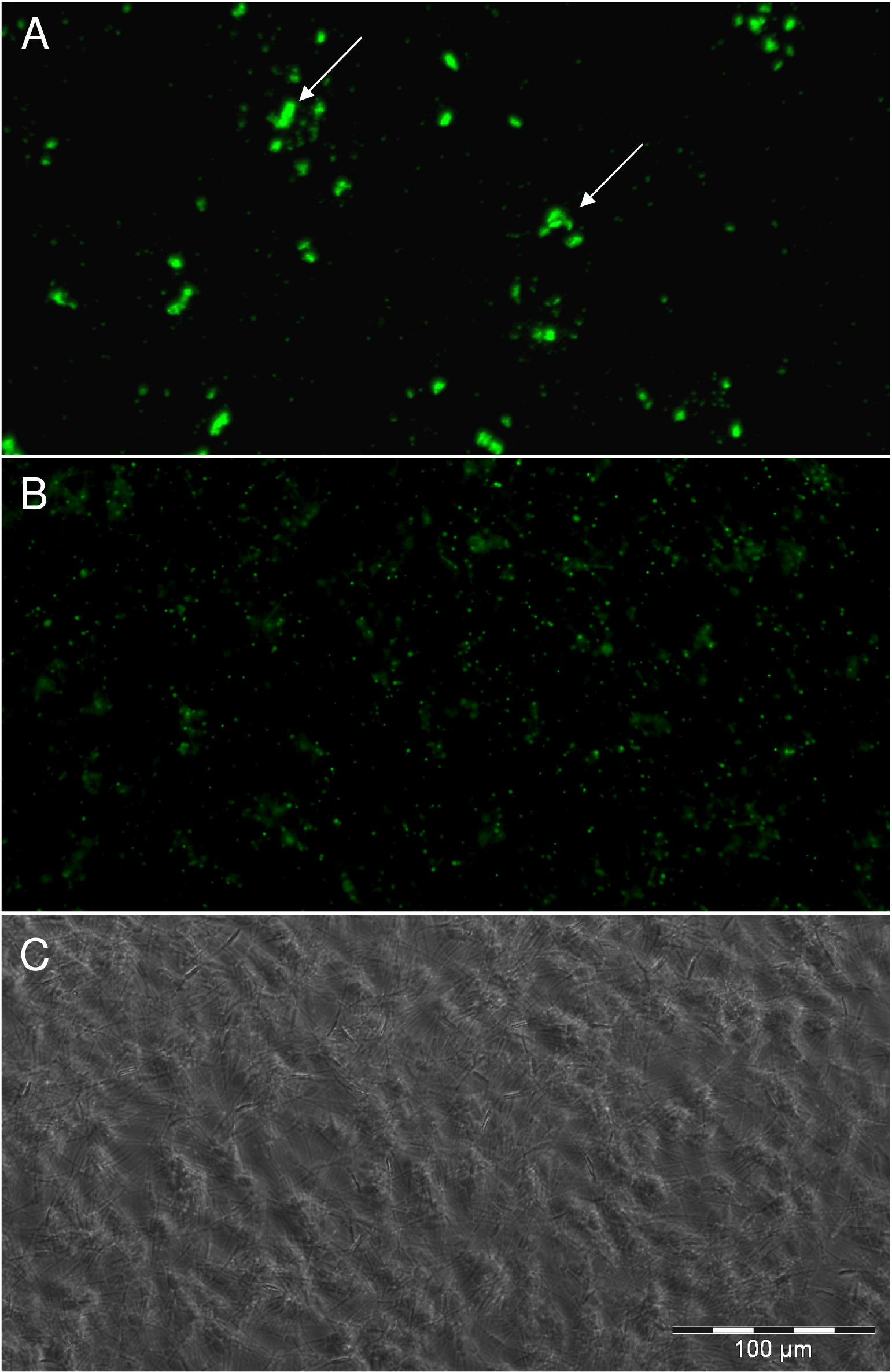

Supplement: Figure S2 — Percoll purification of sperm cells. A) Low magnification view of unpurified Venus-positive spermatozoa. Note the aggregates of somatic cells (epithelial and immune cells), some are labelled by arrows. B) Percoll purified Venus-positive spermatozoa, and C) Brightfield view of B). (TIF) [file pone.0027563.s002.tif]

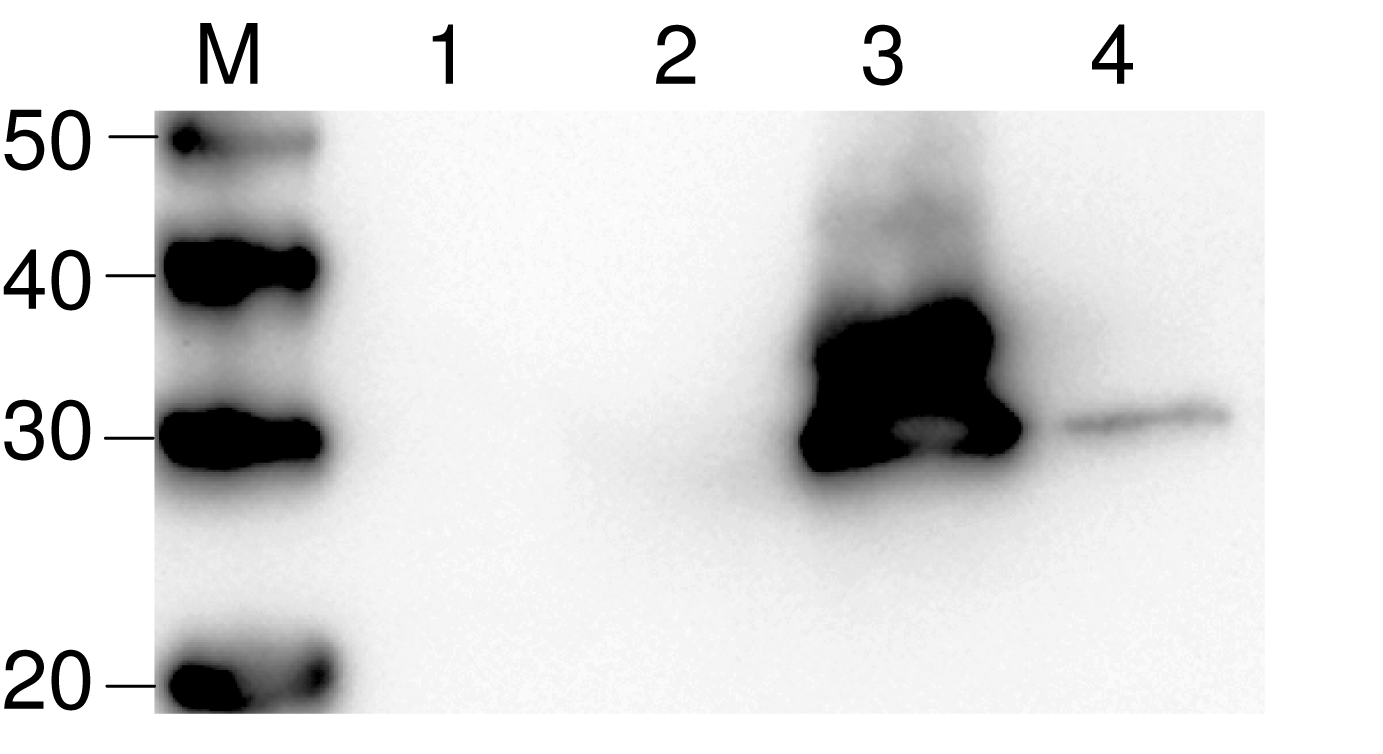

Supplement: Figure S3 — Detection of Venus protein in sperm of transgenic boars. Western blot detection of Venus protein (molecular weight ∼ 30 kilodalton (kD)). Loading of slots: M, molecular size ladder (bands of 20, 30, 40 and 50 kD are indicated); 1-4 protein extracts isolated from: 1, wild type sperm; 2, wild type sperm, Percoll purified; 3, sperm from transgenic boar, 4, sperm from transgenic boar after Percoll purification. (TIF) [file pone.0027563.s003.tif]

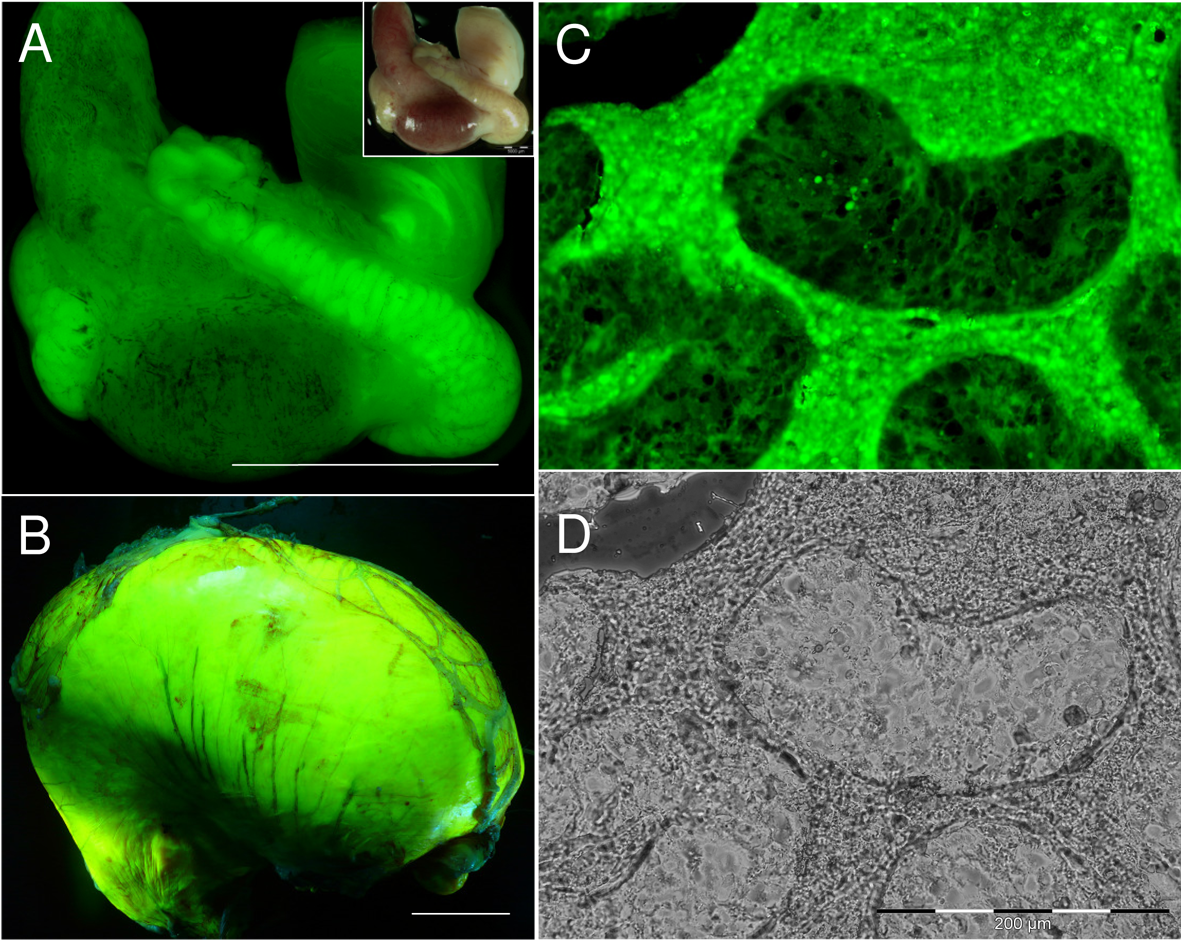

Supplement: Figure S4 — Expression of Venus in transgenic testis. A) Specific Venus fluorescence in testis and accessory glands of a transgenic F1 piglet (day 7 postpartum), which succumbed to a bacterial infection, is shown. Inset, same view under brightfield conditions. B) Venus fluorescence in adult testis (18 months F0 boar). Bars = 2.5 cm. C) Venus fluorescence in cryosection of boar testis and D) corresponding brighfield view. (TIF) [file pone.0027563.s004.tif]
