# Supplementary material for: Genotype-Independent Transmission of Transgenic Fluorophore Protein by Boar Spermatozoa
Source: PLoS One. 2011 Nov 16;6(11):e27563. doi: 10.1371/journal.pone.0027563 (PMC3217978; doi:10.1371/journal.pone.0027563)
Supplement: Table S1 — Primer pairs used for RT-PCR. (DOC) [file pone.0027563.s005.doc]

Supplementary Table 1. Primer pairs used for RT-PCR

| Primer | Sequence | Annealing temperature | No. of cycles,  amplicon length | | | Reference |
| --- | --- | --- | --- | --- | --- | --- |
| Protamine-P1-u | 5´-tcaccatggccagatacagat | 56^o^C | 36, 184 bp | | | [50] |
| Protamine-P1-l | 5´- agtgcggtggtcttgctact |  |  | | |  |
| CD45-u | 5´-agaatactggccgtcgatgg | 56^o^C | 38, 238 bp | | | [50] |
| CD45-l | 5´-gctgaacgcattcactctcct |  |  | | |  |
| YFP1 (Venus1) | 5´-tagcccagggtggtcaccag | 63^o^C | 36, 281 bp | | | [17,18] |
| YFP2 (Venus2) | 5´-tgtgaccggcggctctagag |  | |  |  | |
